# The Role of Structural Polymorphism in Driving the Mechanical Performance of the Alzheimer's Beta Amyloid Fibrils

**DOI:** 10.3389/fbioe.2019.00083

**Published:** 2019-04-24

**Authors:** Gianvito Grasso, Martina Rebella, Umberto Morbiducci, Jack A. Tuszynski, Andrea Danani, Marco A. Deriu

**Affiliations:** ^1^Istituto Dalle Molle di studi sull'Intelligenza Artificiale, Scuola Universitaria Professionale della Svizzera Italiana, Università della Svizzera Italiana, Manno, Switzerland; ^2^Polito BioMEDLab, Department of Mechanical and Aerospace Engineering, Politecnico di Torino, Turin, Italy; ^3^Department of Physics, University of Alberta, Edmonton AB, Canada

**Keywords:** biomechanics, amyloid fibrils, molecular dynamics simulations, Alzheimer's Disease, Young Modulus, structural polymorphism

## Abstract

Alzheimer's Disease (AD) is related with the abnormal aggregation of amyloid β-peptides Aβ_1−40_ and Aβ_1−42_, the latter having a polymorphic character which gives rise to U- or S-shaped fibrils. Elucidating the role played by the nanoscale-material architecture on the amyloid fibril stability is a crucial breakthrough to better understand the pathological nature of amyloid structures and to support the rational design of bio-inspired materials. The computational study here presented highlights the superior mechanical behavior of the S-architecture, characterized by a Young's modulus markedly higher than the U-shaped architecture. The S-architecture showed a higher mechanical resistance to the enforced deformation along the fibril axis, consequence of a better interchain hydrogen bonds' distribution. In conclusion, this study, focusing the attention on the pivotal multiscale relationship between molecular phenomena and material properties, suggests the S-shaped Aβ_1−42_ species as a target of election in computational screen/design/optimization of effective aggregation modulators.

## Introduction

A number of neurodegenerative disorders, including Alzheimer's disease (AD) are related with the abnormal aggregation of polypeptide chains (Chiti and Dobson, 2006). Among several theories proposed to explain the cause of AD, the amyloid hypothesis represents one of the most likely scenarios (Hardy and Higgins, 1992; Reitz, 2012). More in detail, the amyloid hypothesis relates mutations on amyloid precursor proteins (APPs) to the aggregation of Aβ peptides, whose deposition into senile plaques is followed by the formation of neurofibrillary tangles and neuronal cell death (Reitz, 2012). However, if the formation of these fibrils is the cause or a secondary effect of the disease is not clarified yet (Aguzzi and O'Connor, 2010). With the existence of several species of Aβ peptides, it was also observed, that the most common peptides in the amyloid plaques are the Aβ_1−40_ and the Aβ_1−42_, the latter being the most toxic (Querfurth and LaFerla, 2010). For this main reason, previous experimental studies explored the existence of relationships between the molecular structure of amyloid fibrils and disease onset and severity, and on the brittleness of fibrils assemblies (Guo and Akhremitchev, 2006; vandenAkker et al., 2011; Palhano et al., 2013; Schütz et al., 2015; Wang et al., 2017). Earlier models of Aβ_1−42_ fibrils, showed a U-shaped motif characterized by two β-strands V18-S26 and I31-A42 and stabilized by the intra-chain salt bridge between residues D23-K28 (Luhrs et al., 2005).

Nevertheless, recent experimental studies suggested another possible architecture for Aβ_1−42_, based on a S-shaped arrangement (Schmidt et al., 2015; Xiao et al., 2015; Colvin et al., 2016; Gu et al., 2016; Wälti et al., 2016), explored also in recent computational studies (Xi and Hansmann, 2017; Xi et al., 2018). The S-architecture is characterized by a central β-strand with residues V24-G33 connecting C-terminal and N-terminal β-strand. Interestingly, among AD amyloid species, the Aβ_1−42_ is the only one able to take also a S-shape form and it is also the most toxic one (Querfurth and LaFerla, 2010). The above-mentioned evidences let suppose the fibril architecture playing a role in Aβ_1−42_ fibril proliferation and toxicity.

In this context, the mechanical tests of amyloid fibrils are of high importance, considering the well-known interplay between mechanical performance of the fibril architecture and amyloid proliferation (Adamcik and Mezzenga, 2012; Solar and Buehler, 2012; Fitzpatrick et al., 2013; Yoon et al., 2013; Lee et al., 2014; Choi et al., 2015, 2016). Measurements of the stiffness of these β-sheet-rich protein aggregates have provided conflicting results ranging from soft [10^8^ Pa (Sachse et al., 2010)] to extremely hard [10^10^ Pa (Knowles et al., 2007)] Young Modulus depending on the employed methodology, fiber geometry, and assembly characteristics (Knowles et al., 2007; Sweers et al., 2011, 2012; Adamcik et al., 2012; Paul et al., 2016). Several hypotheses have been proposed in literature to unveil the connection between fibrils stiffness and amyloid pathological proliferation. The mechanical failure of amyloid fibrils was proposed as one of the key factors behind the amyloid proliferation, given the correlation between the fracture properties of amyloid fibrils and the propagation of amyloid diseases (Knowles and Buehler, 2011). Other investigations have highlighted the exceptional rigidity of the amyloid material as a key factor to distort cell membranes and disrupt their function (Fitzpatrick et al., 2013).

In the past, experiments such as atomic force microscopy (AFM) (Carrion-Vazquez et al., 2000; Engel and Gaub, 2008; Hane et al., 2013; Lv et al., 2013) were considered to probe *in vitro* the mechanical properties of amyloid assemblies, e.g., elastic modulus (Kol et al., 2005; Graveland-Bikker et al., 2006; Guo and Akhremitchev, 2006; Smith et al., 2006; del Mercato et al., 2008) or bending rigidity (Knowles et al., 2007).

However, nanoindentation or force spectroscopy experiments do not allow to distinguish among U- or S-shaped architecture, structural transitions in polypeptide chains, and interactions governing the mechanical strength of amyloid structures (Raman et al., 2007). Hence the limited resolution of experiments does not allow to clarify the structural composition of the tested amyloids. In this scenario, molecular modeling (Liu et al., 2006; Paciello et al., 2011; Paparcone and Buehler, 2011; Deriu et al., 2012; Havelka et al., 2014; Bidone et al., 2015; Grasso et al., 2015, 2016; Janaszewska et al., 2018), due to their atomic resolution, may help to (1) increase knowledge and (2) rationalize available experimental data. In particular, SMD allows to precisely control the manner in which the force is virtually applied to the system and to clarify the nature of the relationships linking mechanical properties of the system to interatomic interactions (Ndlovu et al., 2012; Solar and Buehler, 2014). Recently, SMD simulations have been employed to evaluate the force needed to pull away a single peptide from U-shaped Aβ fibrils (Raman et al., 2007). *In silico* approaches have also been applied to estimate the Young's modulus of amyloid fibrils (Paparcone et al., 2010), and to examine the dependence of their mechanical stability on the amino acid sequence (Ndlovu et al., 2012).

From the body of available literature, it emerges the need to clearly identify those factors governing fibril material features at molecular level (Sweers et al., 2012), as amyloid fibrils are characterized by a multiscale nature in which nanoscale phenomena determine macroscale properties (Knowles et al., 2007).

The present study employs steered molecular dynamics (SMD) simulations to provide a detailed mechanical characterization of U- and S-shaped Aβ_17−42_ small fibrils. The computational workflow here employed was already successfully applied for similar system in recent literature (Ndlovu et al., 2012; Paul et al., 2016; Poma et al., 2019).

## Materials and Methods

Two different experimental models of a Aβ_1−42_ species were considered in this work: the U-shaped Aβ_17−42_ [PDB ID: 2BEG (Luhrs et al., 2005)] and the recently resolved S-shaped Aβ_11−42_ [PDB ID: 2MXU (Xiao et al., 2015)]. A pentamer of Aβ_17−42_ was extracted from each of the above mentioned experimentally resolved PDB structures (Supporting Information S1). For each molecular structure, we will refer to S-shaped model and U-shaped model in the following.

### Molecular Dynamics Simulations

Each molecular system was solvated in a 7 nm side dimension cubic box and neutralized by counterions. AMBER99-ILDN force-field (Hornak et al., 2006; Lindorff-Larsen et al., 2010) and TIP3P model (Jorgensen et al., 1983) were employed to define protein topology (Hornak et al., 2006; Lindorff-Larsen et al., 2012) and water molecules, respectively. It is worth mentioning that the AMBER99-ILDN force-field has already been indicated as meaningful to investigate conformational properties of Aβ amyloid systems (Carballo-Pacheco and Strodel, 2017). Each system consisted of about 35,000 interacting particles. The models were firstly minimized by applying the steepest descent energy minimization algorithm. Then, in order to increase the statistics of MD data, five independent replicas of each molecular system, differing in initial atom velocities, were obtained from the minimized system. In detail, for each replica a random velocity taken from a Maxwell-Boltzmann distribution at 300 K was assigned to every atom of the system during a preliminary position restrained MD simulation (100 ps time duration) in NVT ensemble. V-rescale thermostat was applied to keep temperature at 300 K with a time constant of 0.1 ps (Bussi et al., 2007). Then, a second position restrained MD (100 ps time duration) in NPT ensemble was carried out for each system at 300 K (τ = 1 ps) and 1 atm (τ = 5 ps). V-rescale (Bussi et al., 2007) and Berendsen (Berendsen et al., 1984) coupling methods were used as temperature and pressure coupling. Then, an unrestrained MD simulation (100 ns time duration) was run for each replica of both S- and U- fibril models. During each production run, the V-rescale (Bussi et al., 2007) and Parrinello-Rahman (Parrinello and Rahman, 1981) approaches were employed for temperature and pressure coupling, respectively. The LINCS algorithm (Hess et al., 1997) was used to constrain the lengths of all bonds, so as to extend the integration time step to 2 fs. Periodic boundary conditions were applied to all (3) dimensions. The short range Van der Waals (VDW) interactions were cut-off after 1 nm, and long range electrostatic forces were calculated with the Particle Mesh Ewald (PME) method (Darden et al., 1993). GROMACS 5.1.2 package was used for all MD simulations and data analysis (Abraham et al., 2015). The overall structural stability of each model was determined through the calculation of the Root Mean Square Deviation (RMSD) of the C-alpha atoms with respect to the initial configuration of the minimized structure (Supporting Information S2).

### Steered Molecular Dynamics Simulations

**Figure 1:**
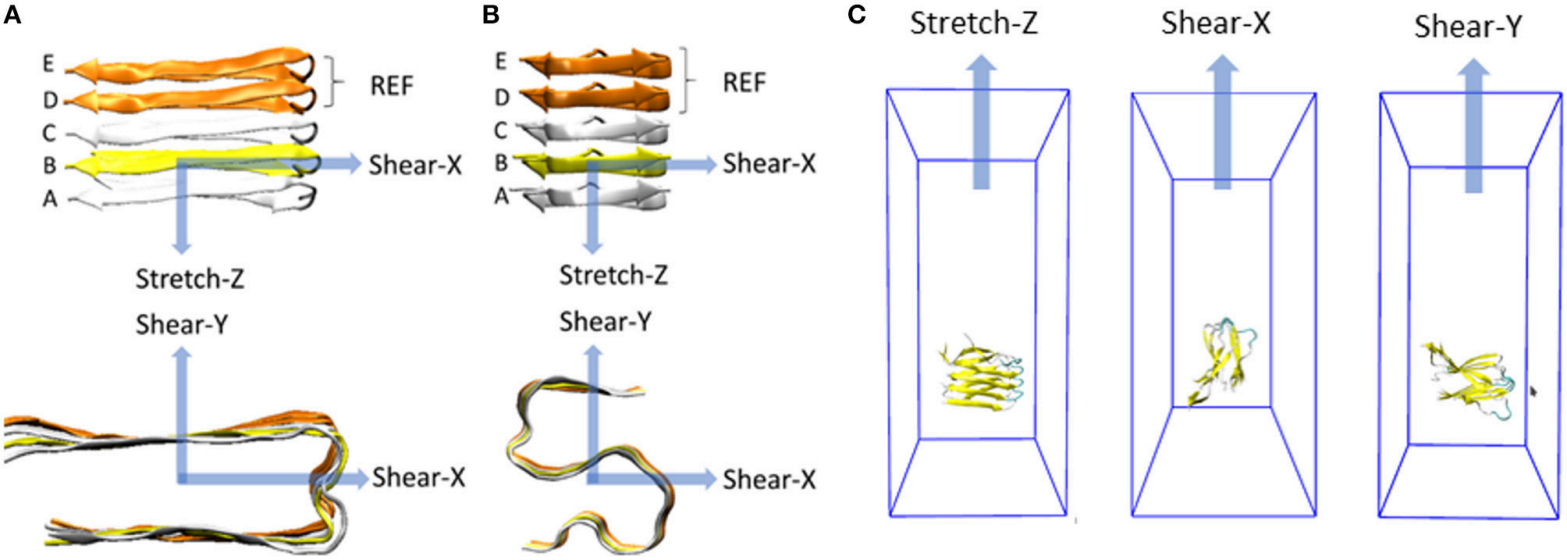
Representation of the deformation protocols applied to the fibril model in case of **(A)** U-shaped and **(B)** S-shaped. Each deformation is defined by a pulling direction, highlighted by the blue arrow. For each deformation, the constrained pulled group and the restrained reference group (both composed only by C-alpha atoms) are evidenced in yellow and orange, respectively. All the other atoms of the peptides are free to move. Molecular systems inside their simulation box throughout stretch and shear SMD simulations are shown in **(C)**.

To compare the mechanical response of S-shaped and U-shaped fibrils, several SMD simulations were performed, differing for the prescribed initial condition represented by a MD output configuration (5 MD replicas for U- and S-shaped models), following the procedure described below (Figure 1).

The fibril axis was aligned with the *Z*-axis in the 3D cartesian space (represented in Figures 1A,B, top row). A defined set of atoms was pulled toward a certain direction while a second group of atoms was position-restrained, as recently done in literature to study the stability of small amyloid fibrils (Ndlovu et al., 2012; Paul et al., 2016; Kouza et al., 2018).

In a greater detail, the C-alpha atoms of the chain B were pulled away from the position-restrained reference group (chain D and chain E). A harmonic force corresponding to the spring constant of 3,000 kJ/(mol nm^2^) was used to pull the chain B away with a velocity of 0.01 Å/ps. Other two pulling velocities (0.1 Å/ps and 0.001 Å/ps) were considered to carefully check the dependence of mechanical response on the choice of pulling velocity (Supporting Information S3).

The V-rescale (Bussi et al., 2007) thermostat was used as temperature coupling. SMD simulations were performed using GROMACS 5 (Abraham et al., 2015).

In summary, 90 SMD simulations have been carried out, i.e., two models (S- and U- fibrils), per five replicas (configurations taken from production dynamics), three pulling directions (*X, Y*, and *Z* as shown in Figure 1) per replica, and three pulling velocities (0.1 Å/ps, 0.01 Å/ps, and 0.001 Å/ps) per each pulling direction.

## Results

### Steered Molecular Dynamics of the Aβ_17−42_ Architectures

**Figure 2:**
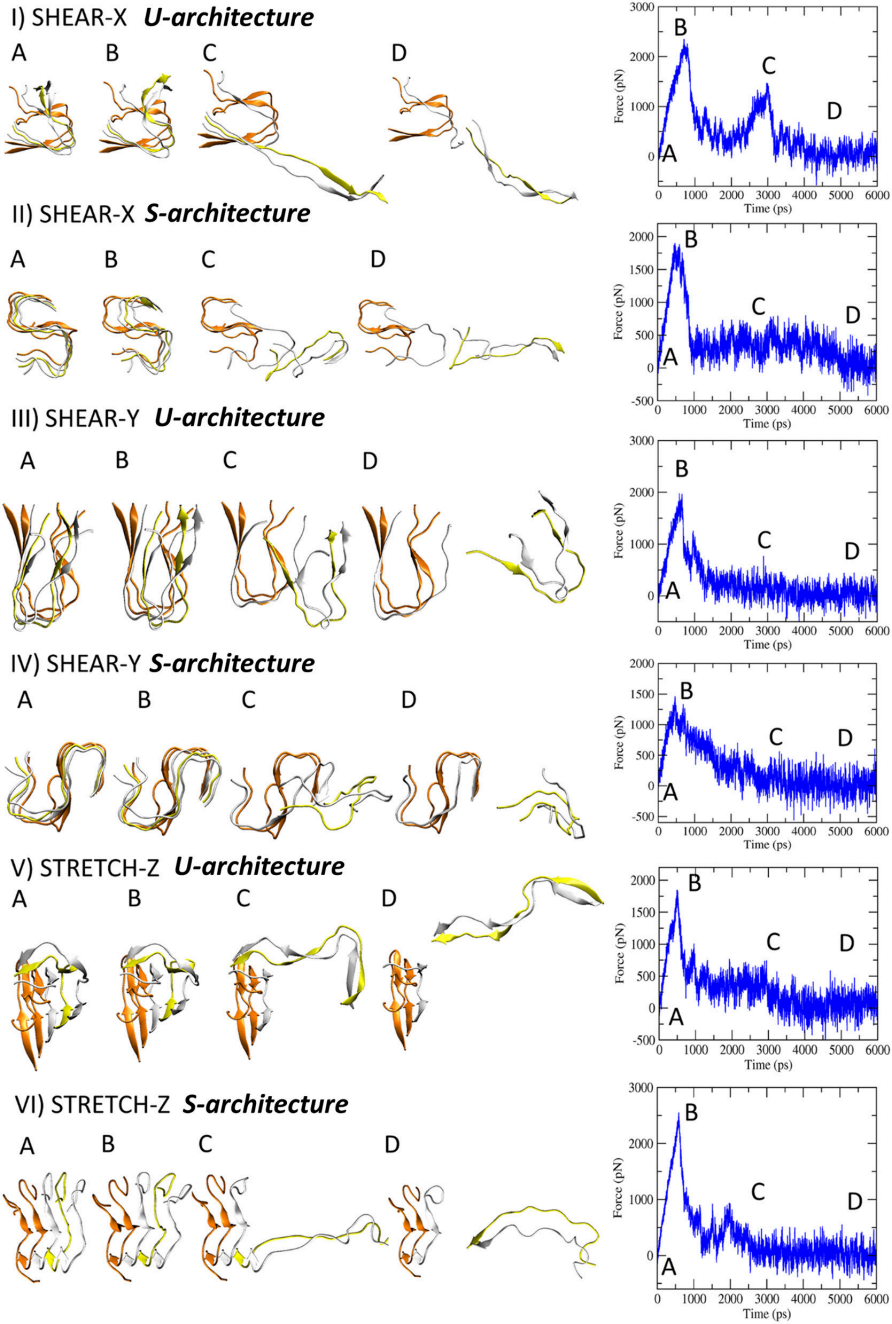
Representative snapshots of U- and S-models evolving in time when the three deformation protocols are applied. On the right side the corresponding force-time profile is plotted.

**Figure 3:**
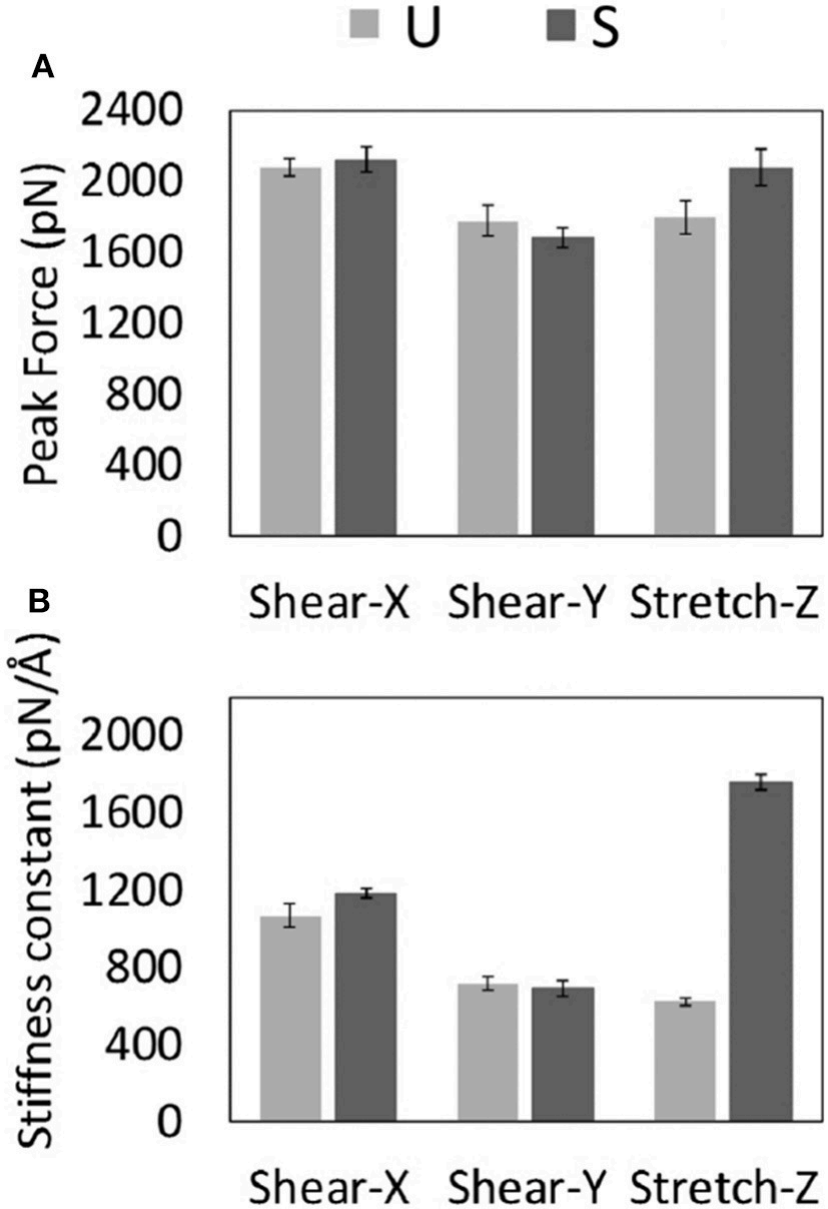
Average values with related standard error of the Peak Forces **(A)** and Stiffness Constants **(B)** recorded for U- and S-models during each type of SMD protocol.

SMD simulations were carried out on the U-shaped model [Aβ_17−42_ pentamer extracted from 2BEG.pdb file (Luhrs et al., 2005)] and the S-shaped model [Aβ_17−42_ pentamer extracted from 2MXU.pdb file (Xiao et al., 2015)] surrounded by explicitly modeled water and ions. All the presented data have been calculated as average ± standard deviation over the 5 SMD replicas for each simulated architecture considering the pulling velocity of 0.01 Å/ps. Other two pulling velocities (0.1 Å/ps and 0.001 Å/ps) were considered to carefully check the dependence of mechanical response on the choice of pulling velocity (Supporting Information S3). More detailed information on simulation set up and analysis are provided in the Method section. Explanatory snapshots taken from representative SMD trajectories, together with the simulated force-time profiles, are presented in Figure 2. The conformational state corresponding to the fibril break and the force [pN] peak indicating the mechanical failure can be clearly identified (Figure 2B). The mechanical resistance of U- and S-shape small fibrils were evaluated in terms of the produced peaks shear (X and Y direction) and stretch (Z direction) force values, averaged over the five replicas (Figure 3A).

A significant difference was observed between stretch force peaks in the two configurations (F_*stretchU*_ = 1.795 ± 186 pN *vs*. F_*stretchS*_ = 2,073 ± 204 pN). On the contrary, data from the other two deformation protocols (*X* and *Y* pulling direction), indicated similar shear force peak values for U- and S- architectures (Figure 3A). It is worth mentioning that the magnitude of forces estimated by SMD is dependent on the imposed pulling velocity (Supporting Information S3), as highlighted by the most current research (Isralewitz et al., 2001a,b). However, the observed difference in the mechanical response to stretching of the two-different fibril U- and S-shaped configurations is also conserved lowering the pulling velocity (Supporting Information S3). Peak forces are in the same order of magnitude of forces calculated in recent literature for similar structures (Kouza et al., 2018).

Analysis of SMD simulations highlighted that the structural anisotropy of amyloid fibrils is likely to manifest in a correspondent anisotropy of their mechanical properties as indicated by quantified stiffness (Figure 3B), i.e., the gradient of the force vs. displacement curves (Supporting Information S3) in the linear response regime (small deformations region). Interestingly, a marked difference between U and S-architectures was observed in the value of the stretching stiffness *k* (*k*_*S*_≈ 3*k*_*U*_), as shown in Figure 3B. The observed shear stiffness (Figure 3B) is lower than stretching stiffness in case of S-architectures, confirming the higher mechanical stability and resistance of the Aβ_17−42_ S-shaped assembly along the fibril direction.

### Mechanical Performance of Aβ_17−42_ Architectures

To provide further insight into the mechanical performance of U- and S-shaped models, the stress-strain curves were computed from SMD force-displacement results along the pulling directions (i.e., X-, Y-, and Z- axis). Technically, each stress value was obtained by dividing the force with the interaction surface between the pulled chain B and the chain C. The application of a linear fitting to the stress-strain data (Figures S5, S6), allowed the estimation of the Young and Shear moduli (*E*,G_xy_, and G_yx_). In detail, the estimated Young's modulus was *E*_*U*_ = 1.4 ± 0.3 GPa and *E*_*S*_ = 2.7 ± 0.3 GPa, for U- and S- architecture, respectively. Moreover, the computed Shear Moduli were G_xyU_ = 0.8 ± 0.3 GPa, G_xyS_ = 1.1 ± 0.3 GPa, G_yxU_ = 0.9 ± 0.1 GPa, G_yxS_ = 0.9 ± 0.2 GPa. An average value of the shear modulus *G* along the *xy* plane (perpendicular to the fibril axis) can be obtained as *G* = (1/*G*_xy_+1/*G*_yx_)^−1^ and results in a value of about 1 GPa for both U and S- architecture.

Noteworthy, the SMD-based estimates of the Young's moduli are in excellent agreement with recent AFM-based experimental data on Aβ_1−42_ species [3.2 ± 0.8 GPa (Adamcik et al., 2012)] and in the range of estimated Young's moduli obtained from other amyloidogenic peptides (Knowles et al., 2007; Sweers et al., 2011, 2012; Adamcik et al., 2012; Paul et al., 2016), as detailed in Table 1. It's also important to highlight that 2- and 3-fold symmetry Aβ_1−40_ fibrils exhibited, as expected, a higher Young Modulus due to a different conformational arrangement and fibril geometry (Xu et al., 2010). It is worth mentioning that no marked variations (in both absolute and comparative values) of the quantified moduli were observed by lowering the SMD pulling rate (Supporting Information S3). The close agreement with experimental data (Knowles et al., 2007; Sweers et al., 2011, 2012; Adamcik et al., 2012; Paul et al., 2016) and the low Young's modulus variation in SMD at lower velocities (0.001 Å/ps) confirm that the proposed simulation set-up is able to properly replicate the mechanical behavior of the investigated fibril configurations.

**Table 1:** Young's moduli of amyloidogenic peptides estimated in literature by previous computational or experimental studies.

**Amyloid species**	**Young Modulus (GPa)**
Aβ_1−42_ species (Adamcik et al., 2012)	3.2
β-lactoglobulin (Adamcik et al., 2010, 2011)	3.3; 4
Aβ_1−40_ (Paparcone et al., 2010; Sachse et al., 2010; Paparcone and Buehler, 2011)	0.05–1.62; 2–18; 18–30
insulin fibrils (Guo and Akhremitchev, 2006)	0.001–0.1
HypF-N protofibril (Relini et al., 2010)	0.06–0.5
hIAPP amyloid fibril (Yoon et al., 2011, 2014)	0.4–0.6; 12–14
α-synuclein fibril (Sweers et al., 2011)	1.2
prion fibrils (Moduli et al., 2014)	0.5–1.3
HET-s prion fibril (Yoon et al., 2013; Solar and Buehler, 2014)	1.5–9.8

### Interatomic Interactions Drive the Mechanical Response Along the Fibril Axis

**Figure 4:**
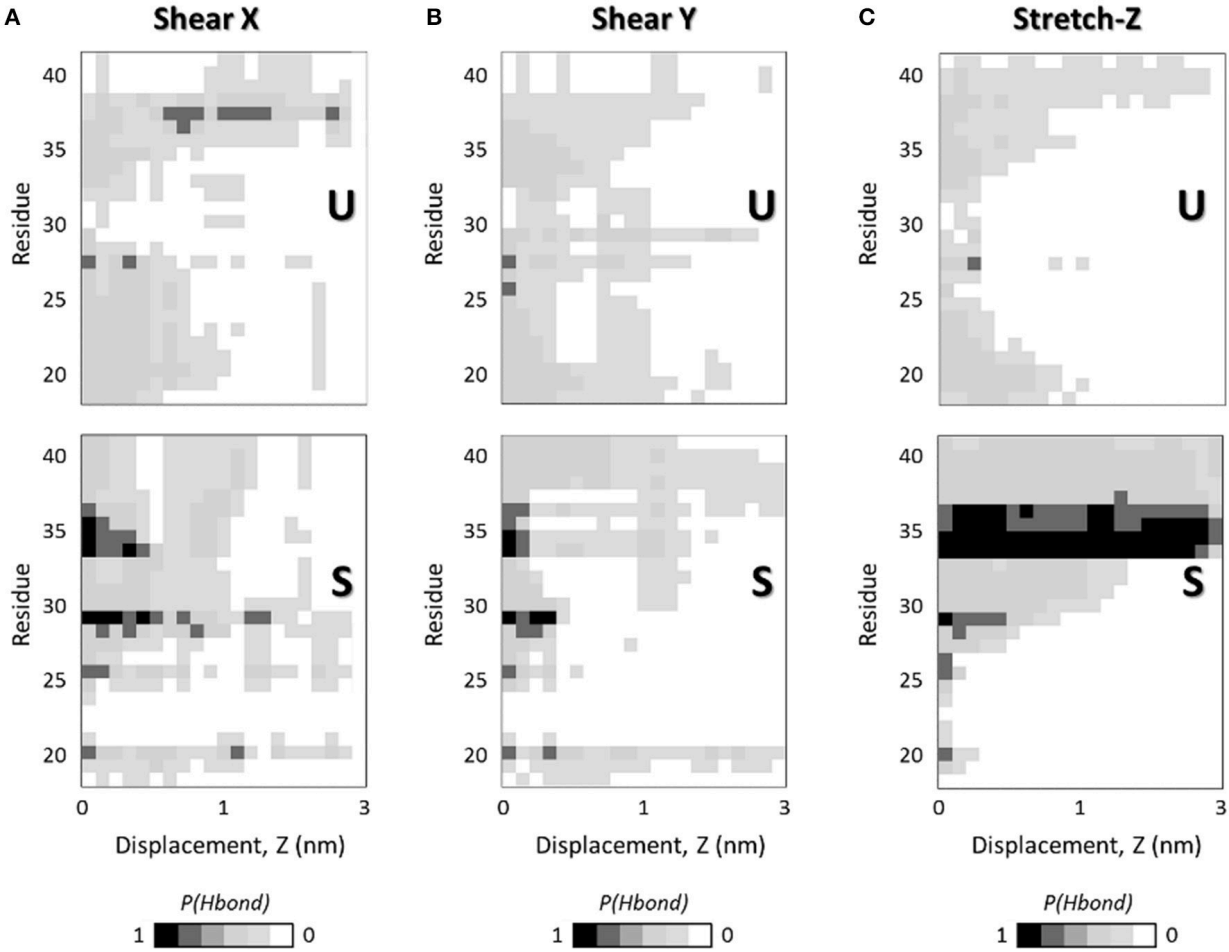
Interchain H-bonds (between chain B and chain C) as function of the interchain chain displacement in **(A)** X-, **(B)** Y-, and **(C)** Z-direction along the SMD simulations at pulling rate v = 0.01 Å/ps. Plots show the probability of H-bonds presence as function of interchain displacement in case of U- (top) and S-shaped (bottom) arrangements. For each residue in one chain (e.g., chain B) the presence of H-bonds with the adjacent chain (e.g., chain C) is quantified as a probability calculated over 5 SMD replicas. Black color indicates a probability of 1, whereas the white code represents a probability of 0, as reported in the legend.

There is ample evidence that the mechanical response of amyloid fibrils is driven by the collective rupture behavior of hydrogen bonds sustaining the cross-β structure of Aβ (Ndlovu et al., 2012; Solar and Buehler, 2014). In particular, the Aβ fibrils are stabilized by a network of backbone hydrogen bonds that acts as a chemical glue allowing them to withstand mechanical forces (Knowles et al., 2007). Here, to provide a deeper understanding of how the interatomic interactions rule the overall mechanical properties of U- and S-shaped fibrils, the hydrogen bonds between the pulled chain B and the chain C were studied in detail. In Figure 4 it is presented how, in both the two configurations, the percentage number of H-bonds between the pulled chain B and chain C along the pulling directions, i.e., X-, Y-, and Z-. The U- and S-shaped configurations during the simulated pulling reacted differently to the applied force (Figure 4). In particular, the S-shaped configuration exhibited a higher tendency than the U-shaped configuration to retain H-bonds as function of the interchain displacement.

The most relevant differences in inter-chain H-bonds between U- and S-shaped configurations are found along fibrils axis (Figure 4C), in agreement with the marked difference observed in the mechanical response during the stretching protocol.

Given that the two fibril configurations share the same sequence, these findings clearly suggest that the different architecture is responsible for eliciting a different chain detachment pathway. In case of U arrangement, the first rupture stage was associated with the disruption of the central H-bonds located at Ala30-Ile31 region. Instead, the S-architecture presented a completely different response, with the N-terminal residues initially (displacement value lower than 1 nm) detached due to the disruption of β_1_ (Ala21-Asp23). For a more clear interpretations of the data, it is worth considering that the per-residue H-bonds depicted in Figure 4 are presented in terms of probability distribution, calculated by sampling all the SMD replicas per architecture, to meaningfully improve the statistical significance of the performed analysis.

## Discussion

Amyloid protein fibrils are a broad class of misfolded protein structures whose mechanical properties are driven by the non-covalent interactions among (primarily) β-sheets, causing the monomers to stack into fibrils (Schleeger et al., 2013). In particular, amyloid beta (Aβ) assemblies, a subclass of amyloid fibrils, share a cross-β motif in which individual β-strands are oriented perpendicular to the fibril axis, resulting in the formation of a dense hydrogen bonded network which extends along the entire length of the fibril (Jaroniec et al., 2004; Sawaya et al., 2007) and gives resistance to the structure.

In the last decade, the intriguing mechanical properties of amyloid fibrils have been the subject of a number of experimental and computational studies (Carrion-Vazquez et al., 2000; Kol et al., 2005; Graveland-Bikker et al., 2006; Guo and Akhremitchev, 2006; Smith et al., 2006; del Mercato et al., 2008; Engel and Gaub, 2008; Adamcik et al., 2012; Ndlovu et al., 2012; Hane et al., 2013; Lv et al., 2013; Paul et al., 2016), motivated by the convincement that investigating the mechanical response of amyloid fibrils could provide crucial insight into many different research contexts.

For example, amyloids have been investigated as possible application in the field of functionalized nanowires, novel edible and biodegradable protein-based polymers (Wei et al., 2017), biological adhesives, and network-type materials (Sweers et al., 2012). Fine tuning of the molecular interactions leading to target mechanical properties of amyloid fibrils represents a pivotal point for further applications of amyloid-based nanostructures as novel biomaterials. Moreover, understanding the physico-chemical properties responsible for to the amyloid mechanical stability may yield an important piece of knowledge into the mechanisms of cellular toxicity. It is now well established that amyloid fibrils are generally polymorphic at the molecular level (Tycko, 2015), with a given peptide or protein being capable of forming a variety of fibrils all having different structural features. This is also the case of the AD, where the Aβ_1−42_ fibril may assume both a U-shaped and S-shaped motif (Schmidt et al., 2015; Xiao et al., 2015; Colvin et al., 2016; Wälti et al., 2016).

Elucidating the connection between the molecular features of a specific fibril architecture and its mechanical performance is therefore crucial to rationally design therapeutic strategies aimed at destabilizing amyloid assemblies.

The different mechanical response of U- and S-shaped architectures was tested here by an extensive computational investigation based on SMD simulations. The resistance of the fibril models to mechanical deformation along specific directions was first analyzed by calculating the magnitude of the mean peak force (Figure 3A). Interestingly the S-shaped arrangement has highlighted better mechanical performances with respect to the U-shaped fibril arrangement. This is an interesting finding, considering that stretching along the fibril axis directly probes the strength of inter-β-sheet hydrogen-bonds. To complete the picture, it was observed that in both U-shaped and S-shaped configuration the interchain hydrogen-bonding network provided a cooperative resistance. As detected by peak force analysis, the S-shaped configuration performed better than the U-shaped one, in terms of stiffness constant along the fibril axis (Figure 3B) and in terms of Young's modulus (E_S_ = 2.7 ± 0.3 GPa, E_U_ = 1.4 ± 0.3 GPa). Notably, these results are in excellent agreement with recent AFM experimental data on Aβ_1−42_ species [3.2 ± 0.8 GPa (Adamcik et al., 2012)], which is exactly the one considered in this work, and in the range of Young's moduli from other amyloidogenic peptides (Knowles et al., 2007; Sweers et al., 2011, 2012; Adamcik et al., 2012; Paul et al., 2016), as detailed in Table 1. As expected, the 2- and 3-fold symmetry Aβ_1−40_ fibrils exhibit a higher Young Modulus due to a different conformational arrangement and fibril geometry (Xu et al., 2010).

The previously mentioned results describe the importance of size effects in elucidating the mechanical properties of amyloid fibrils, as shown in case of different amyloid species (Pampaloni et al., 2006; Xu et al., 2010; Yoon et al., 2011, 2013; Choi et al., 2015). This issue is of great importance for understanding the molecular mechanism behind amyloid growth and proliferation, and it represents a fundamental piece of knowledge to compare computational and experimental results concerning mechanical performance of biological material.

Starting from the well consolidated knowledge about the link between mechanical properties of fibrils and the collective rupture behavior of hydrogen bonds that sustain the cross-β structure of Aβ complexes (Solar and Buehler, 2014), here, a picture of H-bond rupture as function of fibril deformation was also provided, in order to identify the molecular phenomena responsible for the greater mechanical stability of the S-architecture (Figure 4). The most relevant differences in inter-chain H-bonds between U- and S-shaped configurations are found along fibrils axis (Figure 4C). Although the two models are characterized by the same number of H-bonds in the starting configuration, a different reaction to the applied deformation was observed during the stretching dynamics, with the S-architecture H-bond distribution assuring a better distribution of the stress over the interchain surface, and consequently a higher mechanical resistance.

It worth mentioning that, in this work, for the sake of a meaningful comparative analysis, the same protein region (residues L17-A42) has been considered for both U-shaped and S-shaped models. The neglected domain is unstructured and not present in the U-shaped experimental model [2BEG (Luhrs et al., 2005)]. Instead, the experimental S-shaped model [2MXU (Xiao et al., 2015)] contains an additional structured region (residues E11-K16). However, the presence of the E11-K16 structured domain may be reasonably expected to stabilize the interchain hydrogen bonds of protein region L17-V24, further increasing its superior mechanical performance revealed in this manuscript. An evidence is given by Figure 4, indicating the hydrogen bond rupture as function on fibril strain. Whereas, U-architecture rupture always begins in the central area (Ala30-Ile31), the S-architecture disruption starts at N-terminal tail (residues L17-V24) and the unrolling endures progressively to the C-terminal region. Nevertheless, it was previously proved that the presence of the E11-K16 structured domain provide additional interchain hydrogen bonds in the protein region L17-V24 of the S-shaped assembly (Grasso et al., 2018).

Outcomes of the present work indicate that different fibrils architecture results in a different mechanical behavior alog the fibril axis. More in detail, findings point out the attention on the S-architecture, indicating that the mechanical performance is markedly superior to the U-shaped architecture, at least for the case of small fibrils. Apart from the scientific interest in amyloid biomechanics, this work may also stimulate further research on amyloid aggregation modulators. Further studies might consider indication provided in this work for screening compounds able to destabilize the Aβ_1−42_ assemblies, also considering the S-architecture as a target. Based on the excellent agreement with previous experimental data, the SMD simulation-based approach presented in this work is a candidate of election as affordable benchmark platform for screening the efficiency of potential aggregation inhibitors aimed at destabilizing/reducing the stability of the Aβ fibrils.

## Conclusions

In the present work, molecular modeling has been employed to investigate and compare the mechanical performance of the two different architectures proposed to describe the shape of Aβ_1−42_ species_._ Our results provided a clear evidence that the recently proposed S-shaped architecture shows superior mechanical properties with respect with the U-shaped motif due to a better distribution of the mechanical stress along the S-shaped motif. Moreover, the observed findings and the excellent agreement with experimental data suggest the computational protocol here employed as an affordable tool for future design strategies aimed at destabilizing amyloid fibril for therapeutic applications of Alzheimer Disease.

This work does not pretend to be an exhaustive overview about Aβ fibril mechanics. Conformational stability and mechanical performance of amyloid structures depends on a complex interplay of multiscale phenomena from the single monomer to hierarchically aggregates. Hence, Young's and Shear modulus characterization cannot be enough to provide a complete characterization even for a single structured fibril, which has been the focus of the present work.

In general, computational data from Steered Molecular Dynamics strongly depend on the imposed pulling velocity, which is the main parameter to consider in order to correctly evaluate the obtained as usually done in previous relevant literature [9, 14]. In the present study, three different velocities have been applied to carefully check the dependence of mechanical response on the choice of pulling velocity, showing that no marked variations (in both absolute and comparative values) of the quantified moduli were observed by lowering the SMD pulling rate under 0.01 A/ps.

It is worth highlighting that, for the sake of a meaningful comparative analysis, the same amino-acid sequence (residues L17-A42) has been considered for both U-shaped and S-shaped models, as done in our previous study [11]. Whereas, the ignored protein domain is known to be unstructured and not present in the U-shaped pdb file (2BEG [12]), the S-shaped pdb model (2MXU [13]) contains an additional beta-sheet domain between residues E11-K16. The presence of the E11-K16 structured domain is reasonably expected to strengthen the interchain hydrogen bonds, further increasing the stability of the S-shaped fibril assembly. This evidence has been already demonstrated in recent literature [11].

Further investigations are needed in order to better describe U-shaped and S-shaped fibrils rupture dynamics, when a consistent part of hydrogen bonds starts to be broken. An interesting avenue for future studies could be the application of the computational platform developed in the present work to study the destabilization of U-shaped and S-shaped complexes with novel compounds, since despite extensive studies, drug after drug aimed at targeting Aβ has failed the clinical trials.

## Author Contributions

MD and GG conceived the research. GG and MR did the molecular dynamics simulations. MD, GG, JT, UM, and AD analyzed and rationalized the data. All authors wrote the paper and critically commented to the manuscript for important intellectual content, read and approved the final manuscript.

### Conflict of Interest Statement

The authors declare that the research was conducted in the absence of any commercial or financial relationships that could be construed as a potential conflict of interest.

## Abbreviations

Aβ: Amyloid Beta
AD: Alzheimer's disease
AFM: Atomic Force Microscopy
MD: Molecular Dynamics
RMSD: Root Mean Square Deviation
PDB: Protein Data Bank
SMD: Steered Molecular Dynamics.
