# Supplementary material for: The Role of Structural Polymorphism in Driving the Mechanical Performance of the Alzheimer's Beta Amyloid Fibrils
Source: Front Bioeng Biotechnol. 2019 Apr 24;7:83. doi: 10.3389/fbioe.2019.00083 (PMC6499180; doi:10.3389/fbioe.2019.00083)
Supplement: Supplementary file 1 [file Data_Sheet_1.PDF]

Supporting Information

S1 Investigated Models

Two different $A\beta_{1-42}$ models were considered in this work, as reported below.

A) the U-shaped model a pentamer of $A\beta_{17-42}$ extracted from 2BEG.pdb file (Figure S1A)
B) The S-shaped model: a pentamer of $A\beta_{17-42}$ extracted from 2MXU.pdb file (Figure S1B)

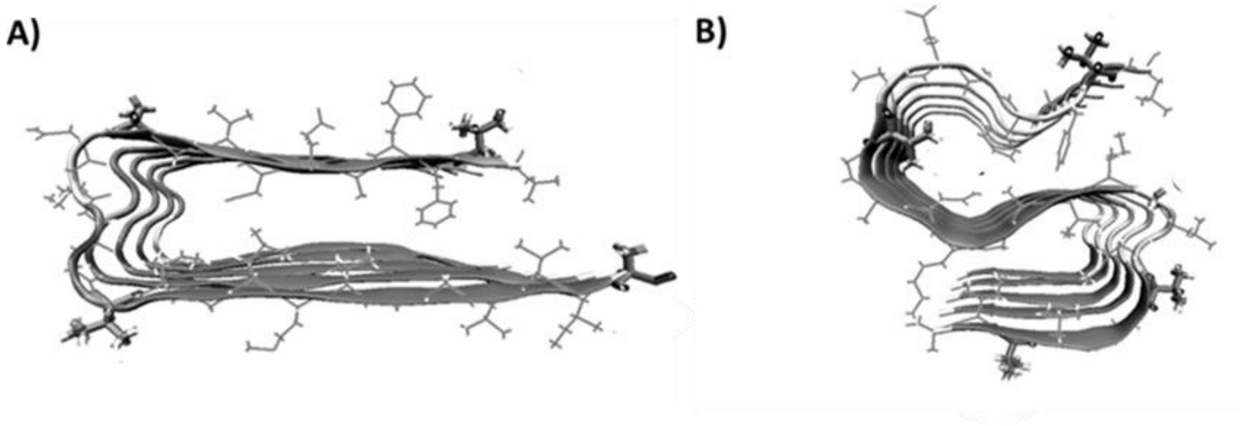

Figure S1: PDB ID of A) U-shape and B) S-shape motif

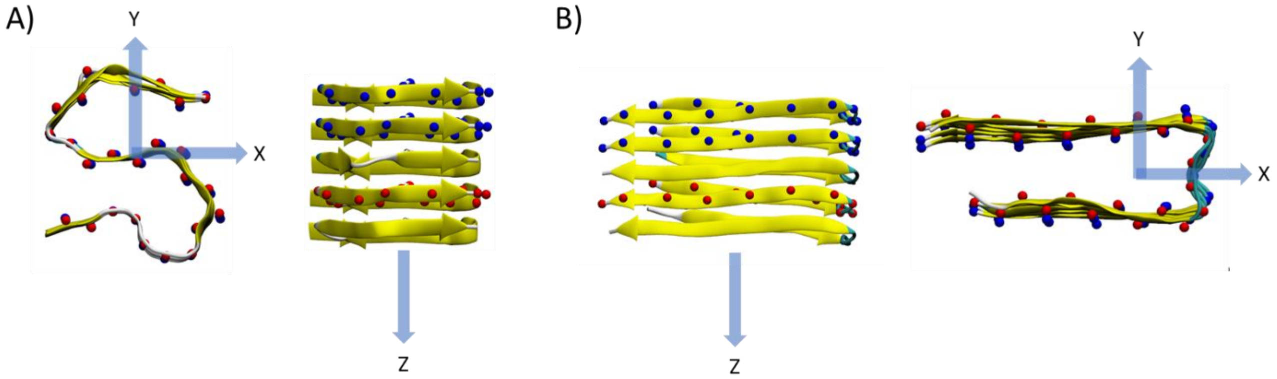

Figure S2. *Representation of the A) U-shaped and B) S-shaped models. Each deformation is defined by a pulling direction, highlighted by the blue arrow. For each deformation, the constrained pulled group and the restrained reference group (both composed only by C-alpha atoms) are evidenced in red and blue, respectively. All the other atoms of the peptides are free to move.*

S2 Conformational Stability

Conformational Stability along all MD simulations was monitored through the Root Mean Square Deviation (RMSD) of C-alpha atoms of each model, as shown in Figure S3. Conformational stability is reasonably achieved in all cases in the last 25 ns.

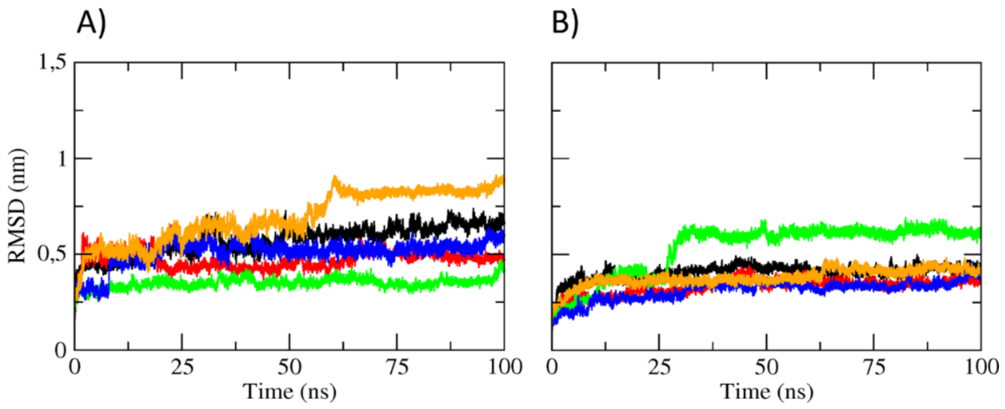

Figure S3: RMSD of C-alpha atoms for the five MD simulations carried out for A) U-shape and B) S-shape structures.

S3 Mechanical properties of U- and S-architectures

In Figure S4 the mean peak force as a function of three different pulling velocities is reported for both U- and S-architectures, 2BEG and 2MXU models, respectively. SMD simulations have been performed until the pulling chain was completely detached from the reference chain of the fibril. This molecular event is dependent on pulling velocity. Pulling velocities equal to 0.1 Å/ps, 0.01 Å/ps and 0.001 Å/ps were applied to carefully check the dependence of mechanical response on the choice of pulling velocity. Thus, simulations have been truncated at 600ps, 6000 ps, and 60000 ps respectively. Steered Molecular Dynamics were performed in 3 directions (X,Y,Z). For each direction, 5 replicas were run at three different rates for both model. Hence a total of 90 simulations were carried out.

The calculation of the mean peak force is dependent on the pulling velocities. In contrast, the difference between these values for each model subjected to the same deformation remains constant and is independent on the pulling velocities.

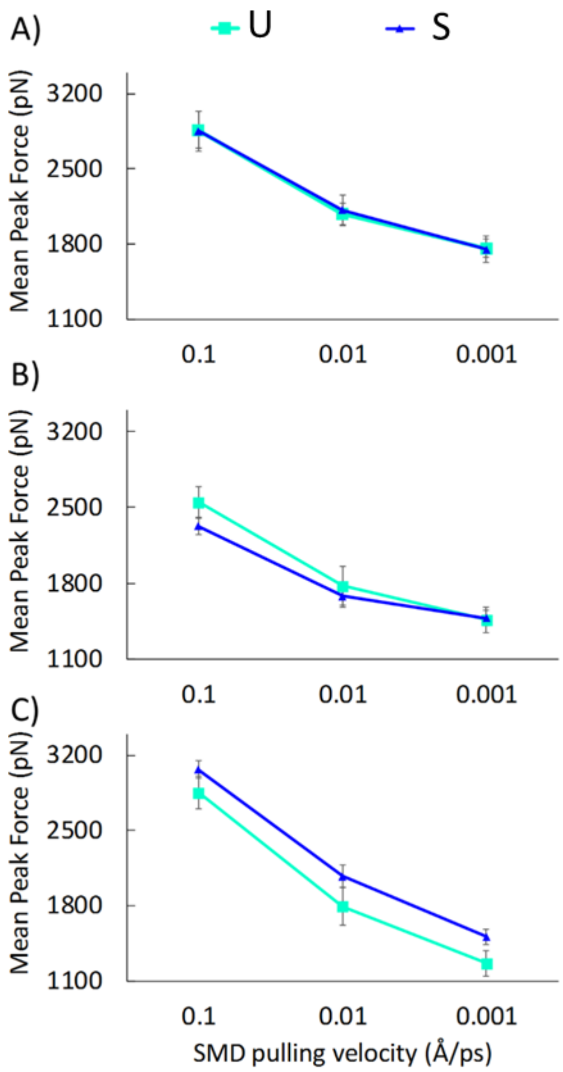

Figure S4: A) mean peak force for the U- and S-model calculated over the A) shear-X, B) shear-Y C) stretch-Z simulations run at three different pulling velocities, with five repeats in each case.

In Figure S5 the force-time profiles measured for U- and S-model in the three pulling directions X, Y and Z (pulling rate 0.01 Å/ps) are shown:

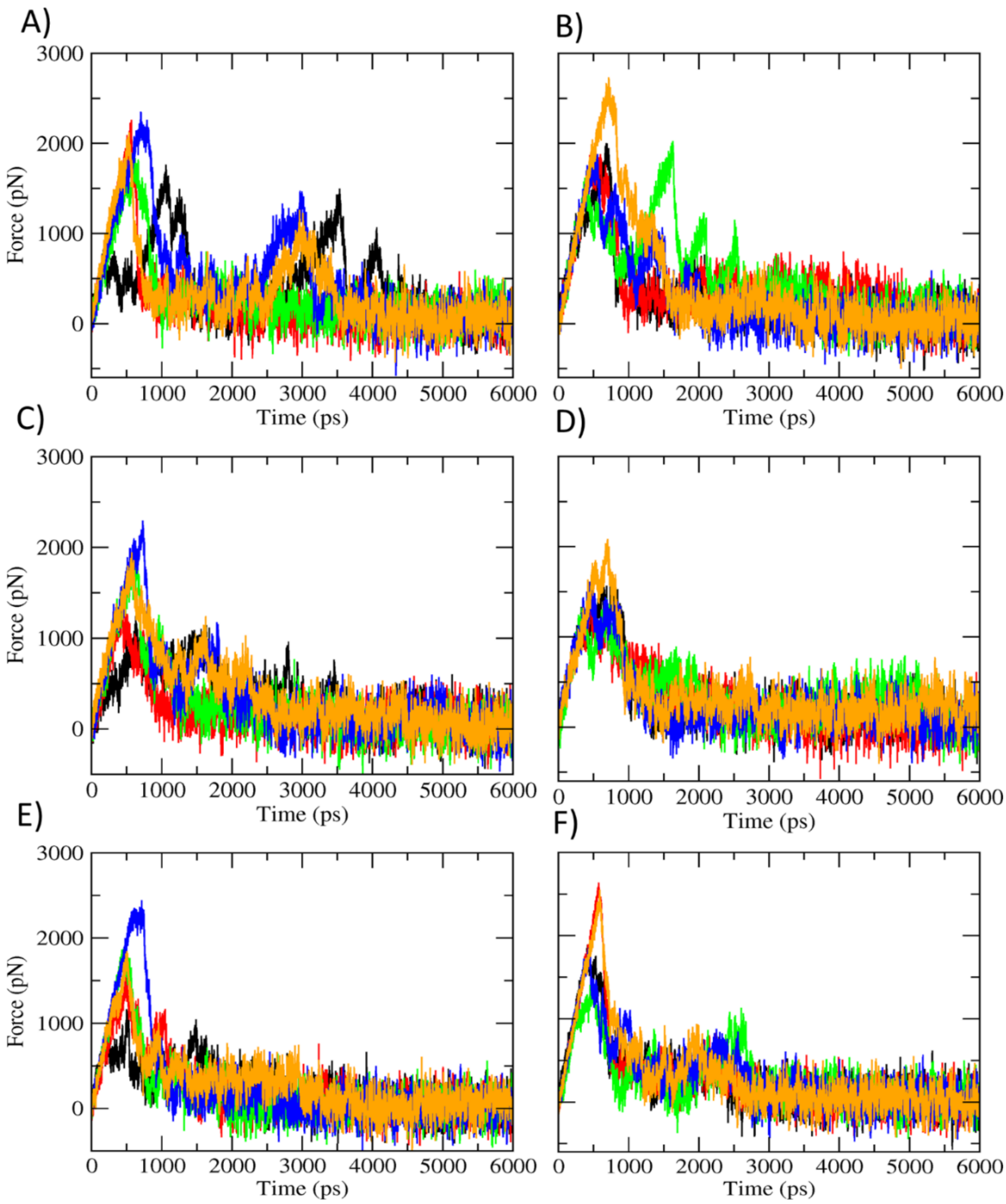

Figure S5: force-time profiles for the U- and S-models, on the left and right panel respectively, as the three different deformations are applied: A) and B) shear-X, C) and D) shear-Y, E) and F) stretch-Z. The colors black, red, blue, green, orange represent the five repeat trajectories carried out for each type of deformation.

Although, as expected, each replica follows a slightly different path through the conformational space, similar features are observed in all equivalent force-time profiles. In Figure S6 the representative force/displacement graphs are reported for each model subjected to the different deformations to show in which area the linear fit (identified with the red line) was performed in order to calculate the stiffness constant.

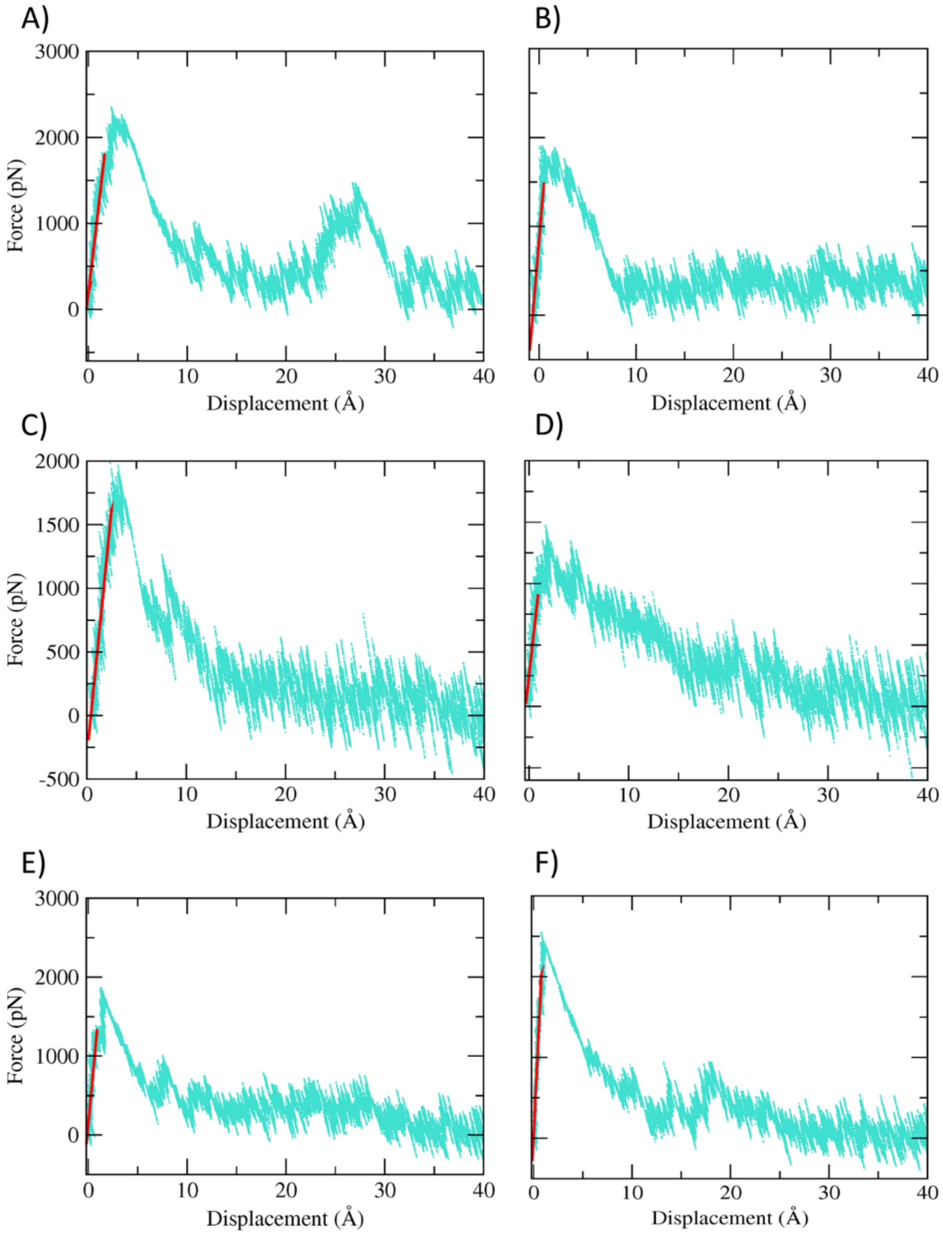

Figure S6. the representative graphs force versus displacement for U- and S-model are reported on the left and on the right side of the panel, respectively. In particular, A) and B) are obtained from the shear-X trajectories, C) and D) from the shear-Y trajectories and E) and F) from the stretch-Z trajectories. The red line represents the linear fit in the elastic regime whose slope is used to calculate the stiffness constant [pN/Å] of the fibril prior to failure.

The stress-strain curves presented in Figure S7 and Figure S8, are obtained from SMD force-displacement results along the pulling directions (i.e., X-, Y-, and Z- axis). Technically, each stress value was obtained by dividing the force with the interaction surface between the pulled chain B and the chain C. The application of a linear fitting to the stress-strain data in Figure S7 and Figure S8, allowed the estimation of the Young and Shear moduli (E,G_x, and G_y) of both the two-fibril configuration. The plot of strain versus time is reported in Figure S11, in case of a representative SMD simulation of S-shaped and U-shaped model subjected to stretching deformation. The figure demonstrated the elastic behavior of the fibril until the detachment of the pulling group (at strain value of about 0.1).

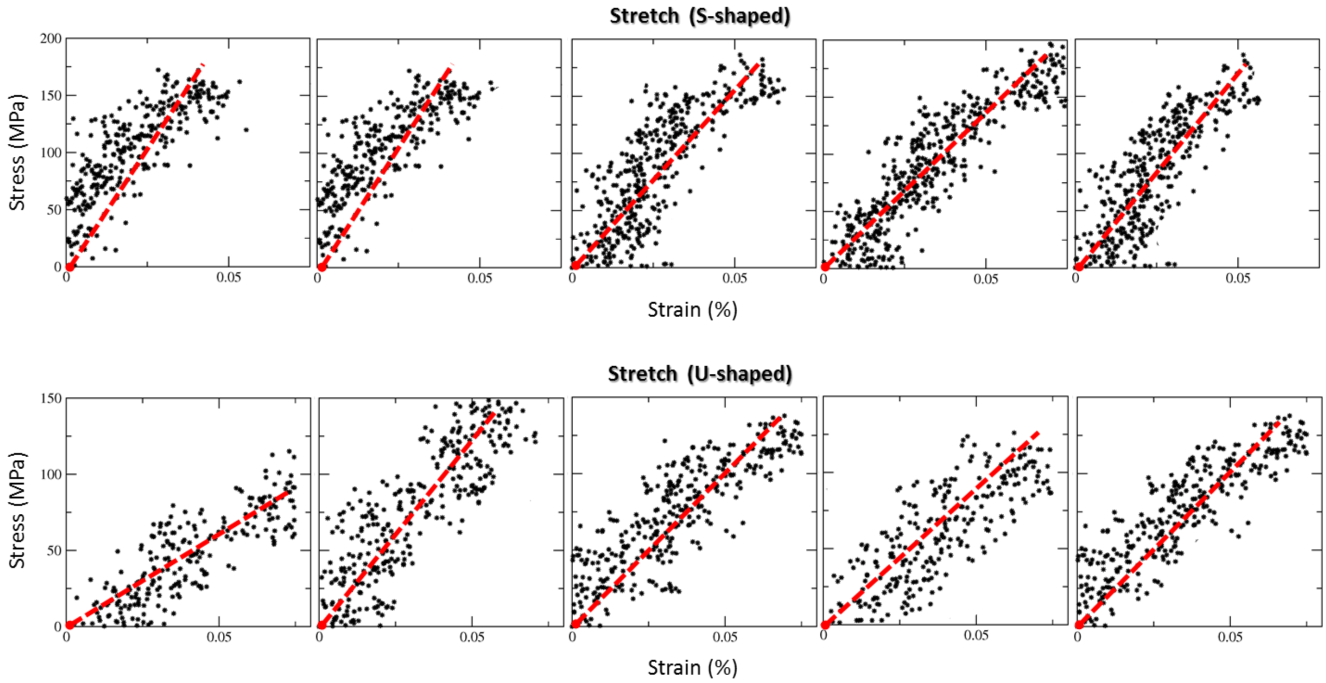

Figure S7. Stress-strain curve obtained from the force-displacement results of the two models subjected to the stretching deformation along the fibril axis (Z-direction). The stress is calculated as (force [pN])/(buried surface [nm^2]). The force is applied along the Z- axis. The buried surface is calculated between the chain B and chain C. The solid line represents the linear fit (correlation coefficient higher than 0.8 in all cases) in the elastic regime and small deformations. The linear fit slope is the Young's modulus [GPa] of the fibrils.

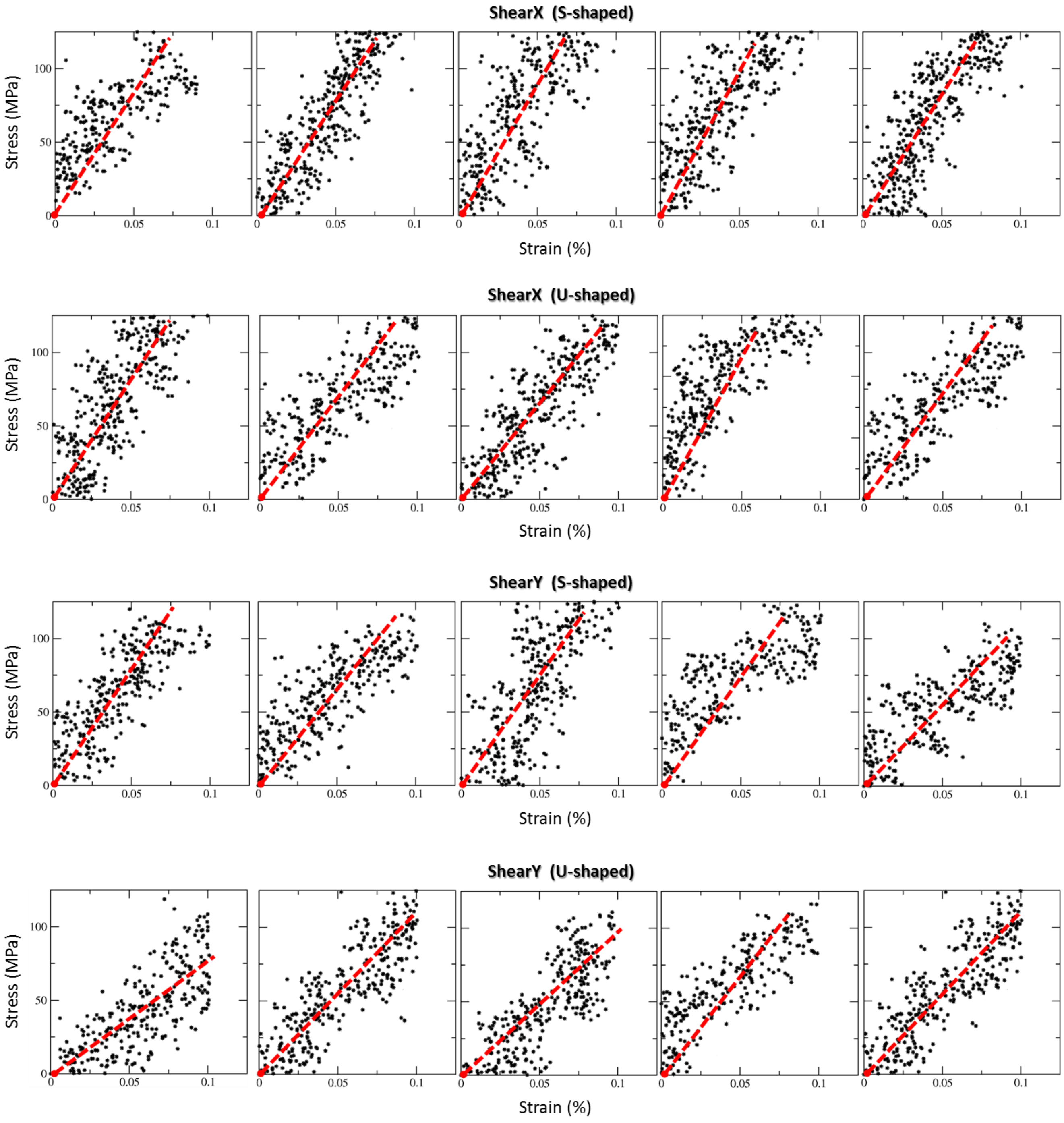

Figure S8. Stress-strain curve obtained from the force-displacement results of the two models subjected to the shear deformation along the X- and Y-direction. The stress is calculated as (force [pN])/(buried surface [nm^2]). The force is applied along the X- and Y- axis, respectively. The buried surface is calculated between the chain B and chain C. The solid line represents the linear fit (correlation coefficient higher than 0.8 in all cases) in the elastic regime and small deformations. The linear fit slope is the Young's modulus [GPa] of the fibrils.

In Figure S9 the Young's modulus as a function of three different pulling velocities is reported for both U- and S-models subjected to the stretching deformation. Lowering the pulling rate from 0.01 to 0.001 Å/ps, we did not detect, on the same model, statistically significant differences of Young's modulus.

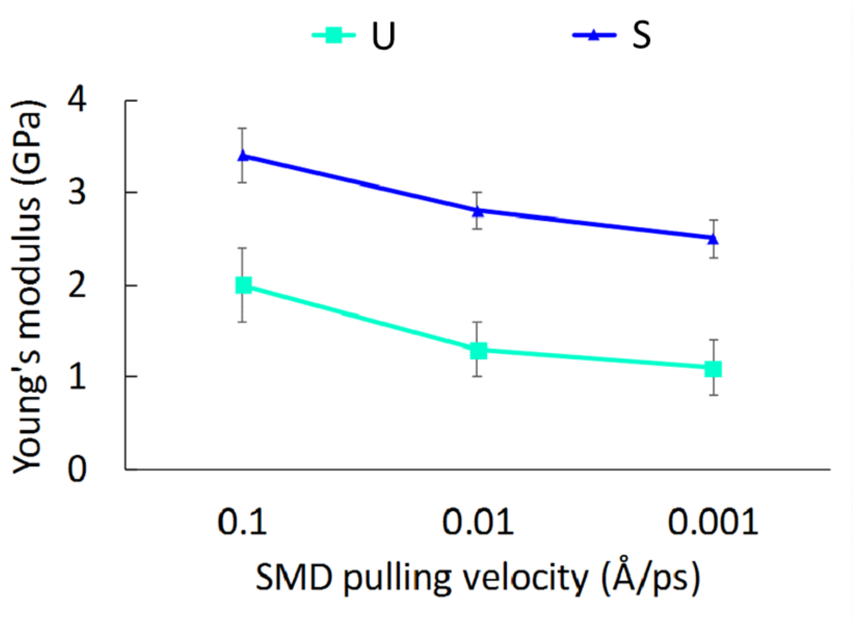

Figure S9. Young's modulus [GPa] for the U- and S-model calculated over the stretch-Z simulations run at three different pulling velocities, with five repeats.

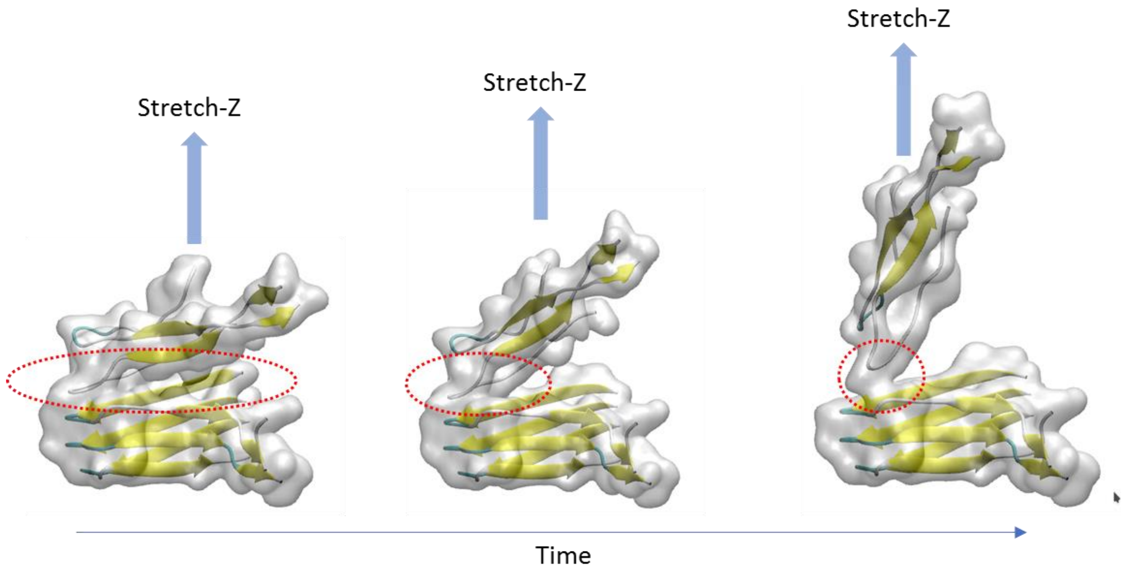

Figure S10. Molecular System along the Steered Molecular dynamics. The red line highlights the contact surface region considered to compute the stress-strain curve.

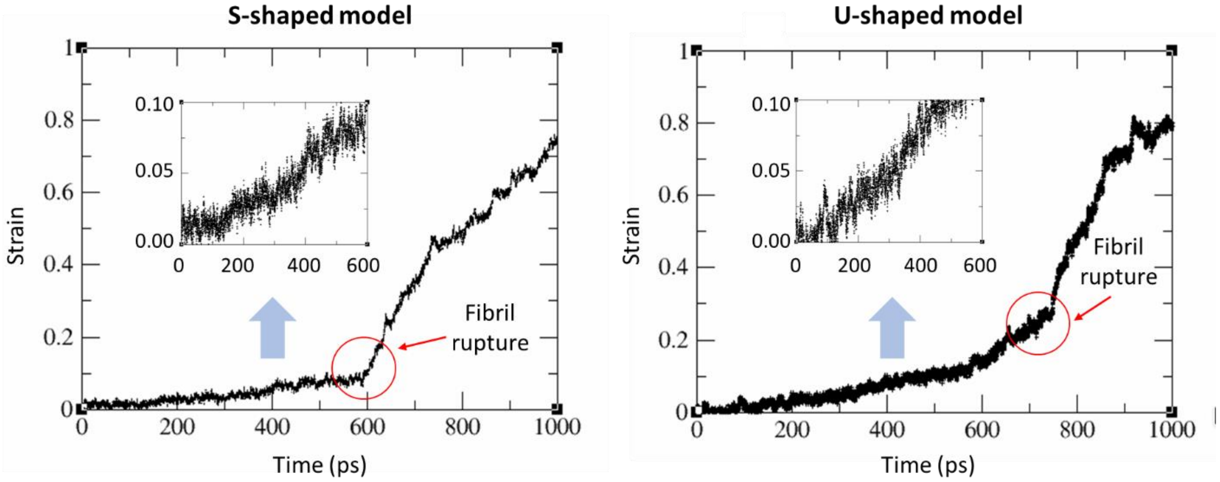

Figure 11. Strain versus time plot in case of a representative SMD simulation of S-shaped and U-shaped fibril subjected to stretching deformation
